# EQ-5D™-derived utility values for different levels of migraine severity from a UK sample of migraineurs

**DOI:** 10.1186/1477-7525-10-65

**Published:** 2012-06-12

**Authors:** Megan R Stafford, Asha Hareendran, Daisy S Ng-Mak, Ralph P Insinga, Ruifeng Xu, Donald E Stull

**Affiliations:** 1 Outcomes Research, United BioSource Corporation, 26-28 Hammersmith Grove, Floor 5E, London, W6 7HA, United Kingdom; 2 NCCMH, Royal College of Psychiatrists, (during the work conducted and preparation of this manuscript, MRS was employed by United BioSource Corporation), 4th Floor Mansell Street, 21 Standon House, London, E1 8AA, United Kingdom; 3 Director, Global Health Outcomes Strategy & Research, Allergan, (during the work conducted and preparation of this manuscript, DSN was employed by Merck Research Laboratories), 2525 Dupont Drive (MI3-110A), Irvine, CA, 92612-1599, USA; 4 Department of Health Economic Statistics, Merck Research Laboratories, Merck Sharp & Dohme Corp, Whitehouse Station, NJ, 08889, USA; 5 RTI-Health Solutions, (during the work conducted and preparation of this manuscript, DES was employed by United BioSource Corporation), 2nd floor, the Pavilion; Towers Business Park, Wilmslow Road, Didsbury, Manchester, M20 2LS, United Kingdom

**Keywords:** Migraine, EQ-5D, MIDAS, Quality of life, Health utility

## Abstract

**Background:**

To estimate utility values for different levels of migraine pain severity from a United Kingdom (UK) sample of migraineurs.

**Methods:**

One hundred and six migraineurs completed the EQ-5D to evaluate their health status for mild, moderate and severe levels of migraine pain severity for a recent migraine attack, and for current health defined as health status within seven days post-migraine attack. Statistical tests were used to evaluate differences in mean utility scores by migraine severity.

**Results:**

Utility scores for each health state were significantly different from 1.0 (no problems on any EQ-5D dimension) (p < 0.0001) and one another (p < 0.0001). The lowest mean utility, − 0.20 (95% confidence interval [CI]: -0.27 – -0.13), was for severe migraine pain. The smallest difference in mean utility was between mild and moderate migraine pain (0.13) and the largest difference in mean utility was between current health (without migraine) and severe migraine pain (1.07).

**Conclusions:**

Results indicate that all levels of migraine pain are associated with significantly reduced utility values. As severity worsened, utility decreased and severe migraine pain was considered a health state worse than death. Results can be used in cost-utility models examining the relative economic value of therapeutic strategies for migraine in the UK.

## Background

Migraine is an episodic but recurrent pain syndrome characterised by neurological and gastrointestinal symptoms, and is associated with impaired functioning, quality of life and psychological impairment [1]. Migraines also lead to a considerable economic burden related to indirect costs (such as absenteeism and reduced work productivity) [2] and direct costs (such as drug treatment, physician costs, diagnostics, and emergency room visits) [3].

Clinical trials of products for the treatment of acute migraine focus on outcomes of headache response according to four levels of severity for migraine pain (none, mild, moderate, and severe); in terms of improvement from moderate or severe to mild or no pain; or improvement to no pain, at particular points in time [4]. To enable health care decision makers to examine the relative value of various treatments available to treat migraine, it is important to understand outcomes in terms of their effects on overall health status, on a common metric. Utility measures can be used to estimate quality-adjusted life years (QALY)—the preferred metric to value improvements in health status for some health care decision makers (e.g., National Institute for Health and Clinical Excellence [NICE] [5]).

A utility measure captures the relative preference or value that an individual associates with different health states, and is scored as a single index where a value of 1 denotes complete health and a value of 0 represents ‘dead’. However, 0 is not necessarily the lower limit of the scale; the lower limit for the EQ-5D in a UK sample is estimated as -0.5 [6]. Utilities can be elicited directly from patients using valuation techniques (such as the standard gamble or time trade-off), or through the use of generic preference-based measures such as the Health Utilities Index (HUI) [7] or EuroQol EQ-5D survey (EQ-5D^TM^ is a trademark of the EuroQol Group) [8-10]. In the UK, NICE recommends the use of the EQ-5D for measuring and valuing health states, and for estimating utility values for use in cost-effectiveness analyses [5].

Despite the extensive literature describing a decreased quality of life associated with a migraine, the temporary nature of a migraine attack has rendered it difficult to capture patients' utility during a migraine attack, as well as any changes in patient utility that might occur within a migraine attack. Very few studies to date have measured utility for migraine pain severity levels and outcomes.

Brown et al. (2008) elicited utilities using the HUI Mark 3 (HUI3) in a United States (US) cohort of migraine patients with a 4-week recall period (potentially covering time within and outside of a migraine episode), and reported that utility was inversely related to headache frequency [11]. Xu et al. (2010) reported the relationship between migraine pain and health utility based on the analysis of clinical trial data on the EQ-5D in a sample of US patients [12]. They estimated the disutility for moderate and severe levels of headache pain as the difference in EQ-5D scores for patients between trial baseline (patients experiencing either moderate or severe pain) and 24-hour time points (if pain free), and found that severe migraine was associated with the greatest disutility. They reported significantly lower utilities (estimated using indirect methods) for mild pain than for no pain (p < 0.0001), suggesting a greater value for treatments that eliminate migraine altogether compared with those that reduce migraine to a mild level.

Luo et al. (2009) compared the ability of the EQ-5D and the HUI Mark 2 (HUI2) and HUI3 index systems to discriminate between the presence or absence of chronic medical conditions, and found differences in utility values obtained for respondents with migraine (sampled either outside of or during an attack) on each of the three instruments (0.82, 0.80, and 0.72, respectively), suggesting utility value elicitation can be affected by the methods employed [13].

To our knowledge, there are currently no studies that have estimated utility values for migraine pain severity levels and outcomes using the EQ-5D in a UK population of migraineurs. The goal of our study was to understand the effect of different levels of migraine pain severity on health statuses and utility values for a UK sample. By using a utility-based metric that is useful for health care decision makers, this information can contribute to health economic evaluations for migraine prevention and treatment. This study is unique, in that it seeks to collect utilities for a recent migraine when migraine patients were not experiencing a migraine or any residual symptoms. In addition, it addresses the need for utilities for migraine pain in the UK.

## Methods

This was a cross-sectional, observational study that enrolled participants who had recently experienced a migraine in the UK. We administered the EQ-5D to generate health utility values for migraine sufferers in the UK for different levels of migraine pain severity for participants’ most recent attack and their current health outside of an attack.

### Participants

Migraine sufferers were recruited through advertisements posted on migraine support group websites in the UK, and by using support groups’ databases. Researchers trained in study procedures screened 166 interested participants via the telephone using a structured clinician-validated screening form, which included study eligibility criteria and, to confirm a diagnosis of migraine (with or without aura), International Headache Society Criteria [14]. Participants were enrolled in the study if they were over 18 years of age, had at least one migraine in the past seven days, and had a history of physician-diagnosed migraine headaches for at least six months. Migraine sufferers with visual or cognitive impairments, as assessed by the researcher and those experiencing residual migraine symptoms from the most recent migraine were excluded. Tension-related component of the headache was not evaluated.

#### Description of questionnaires used

The EQ-5D questionnaire is a self-administered, generic questionnaire designed to assess health status across a wide range of health conditions and treatments. The EQ 5D can be weighted using social preferences (utilities) or scored in several other ways; for example, using visual analogue scale (VAS) ratings to provide a simple descriptive profile. The former method is predominantly used in economic evaluation. The health utility values are based on TTO scores and can be calculated from profile ratings using a scoring algorithm [15]. The EQ-5D is comprised of two sections. The first section consists of five dimensions to assess health-related quality of life (HRQL): mobility, self-care, usual activities, pain/discomfort, and anxiety/depression. Each dimension is rated on a 3-point scale: 1 (no problem), 2 (some or moderate problems), or 3 (unable or extreme problems). The second section consists of a 20 cm vertical VAS, with anchors of 0 (“worst imaginable health state”) and 100 (“best imaginable health state”).

Participants were also asked to record all pain levels experienced during their most recent migraine using a standard 4-grade pain scale [16] (no pain, mild pain, moderate pain, severe pain). They were also asked to respond if they were on treatment for their migraine, or whether they experienced any comorbid conditions on a list of common co-morbidities identified by a clinician. Subjects could also add any other comorbid conditions as free text.

The Migraine Disability Assessment Questionnaire (MIDAS) is a self-administered patient-reported questionnaire aiming to capture headache-related disability, and contains three dimensions: paid work and education (school/college), household work and family, and social and leisure activities. The MIDAS was scored based on the number of days of lost and limited activity due to migraine, with lower MIDAS scores reflecting less headache disability [17]. The MIDAS was scored according to a standard algorithm, resulting in a range of scores that were categorised into four grades of migraine disability:

· Grade I: little or no disability (0-5)

· Grade II: mild disability (6-10)

· Grade III: moderate disability (11-20)

· Grade IV: severe disability (21+)

#### Procedures

At screening, if patients had experienced a migraine headache in the past seven days, and were currently not experiencing any residual symptoms, a study visit was scheduled as soon as possible. If the participant had not experienced a migraine headache in the previous week, he or she was contacted at regular time points to monitor if he or she had experienced a migraine headache. In these instances, a study visit was scheduled within one week of a migraine if no residual symptoms were being experienced.

On the day of their study visit, participants completed the sociodemographic questionnaire (including questions about medications and comorbidities), the MIDAS, and the EQ-5D for their current health (without migraine). Following completion of these questionnaires, participants were queried regarding their utility for mild migraine pain, moderate migraine pain, and severe migraine pain levels within the same recent migraine attack.

To enable recall for each level of pain severity experienced during their recent migraine, participants were instructed to recall their most recent migraine attack using a calendar of the past four weeks. After participants identified the date (or dates) of their most recent migraine, they were provided with the face of two clocks, one for the AM hours and one for the PM hours. Using these props, the participants were asked to reflect on their most recent migraine experience and draw on the calendar and clocks what dates and hours they had experienced their migraine. Participants were next asked to subjectively assess the severity of their migraine pain by using a standard pain scale [16]. They would then categorise the experience of migraine pain severity during their most recent attack as mild, moderate, and/or severe by labelling the dates and hours they had experienced their migraine using these categories.

The participant then completed the EQ-5D for each level of migraine pain severity that they experienced (mild, moderate, or severe). If participants had experienced only one level of severity during their most recent attack, they did not complete the EQ-5D for other levels of severity. If all severity levels were experienced during the attack, participants were asked to complete the EQ-5D for all three levels of severity. Participants were also asked to complete the EQ-5D for their current health state on that day, which was within seven days post-migraine attack, and with no residual migraine symptoms present (i.e., current health without migraine).

Therefore, the maximum number of EQ-5D administrations per participant was four (mild pain, moderate pain, severe pain, and current health [without migraine]), and the minimum number of EQ-5D administrations was two (only one level of severity experienced and current health [without migraine]).

The study was conducted by researchers experienced in conducting the study procedures.

### Data analysis

Descriptive statistics were calculated for sociodemographic and MIDAS data. All EQ-5D data were summarised and grouped according to the migraine state (mild pain, moderate pain, severe pain, and current health [without migraine]), with basic statistics given for the continuous data (number, mean, and SD). Utility values were calculated according to standard procedures, and using the York preference tariff [6].

Pearson's correlation coefficient was calculated to assess whether there was a relationship between migraine-related disability and utility using the participant's MIDAS score and his/her utility for the current health (without migraine) state.

Utility values were compared between severity levels for participants who had non-missing values for both data points in the comparison of interest. Paired t-tests were used to compare mean utility values for mild, moderate, severe, and inter-ictal periods against the inter-ictal values to evaluate whether there were significant differences in mean patient utility by migraine severity. In addition, if the EQ-5D data were not normally distributed, non-parametric analyses (Wilcoxon signed test) were planned.

It was estimated that a sample size of 100 would be sufficient for estimating significant differences in utility scores between four migraine states at a significance level of p < 0.05 and with 90% power to detect significant differences.

Participants were required to report on at least one of the four health states being evaluated (current health [without migraine], mild migraine pain, moderate migraine pain, or severe migraine pain) to be eligible for analysis. In the event of missing responses within a questionnaire, guidelines from instrument developers were used to handle missing data where available [15,17].

## Results

### Participant characteristics

Participant characteristics are shown in Table 1. A total of 106 migraine sufferers were recruited. The mean age of our sample was 47.4 years, with 76% female. The mean length of time since initial migraine diagnosis was 19.2 years. Over half of our sample (52.9%) was prescribed medication to treat migraine. Eighty-four participants (79.2%) reported at least one comorbidity; the number of comorbidities per participant ranged from 1-7. Of the comorbidities listed in the sociodemographic form, the most frequently experienced were depression (33%), followed by anxiety (31%). Congestive heart failure was reported the least frequently (0.9%). Over a third of the sample (34.9%) reported “other” comorbidities that were not otherwise listed.

**Table 1:** Participant Characteristics

**Demographic Attributes**	**N = 106**
**Age (in years), mean (SD)**	47.45 (11.71)
**Gender, N (%)**	
Male	25 (23.6%)
Female	81 (76.4%)
**Ethnicity, N (%)**	
Caucasian/White	89 (83.2%)
Black	6 (5.6%)
Mixed/Other	3 (3.8%)
Asian	8 (7.4%)
**Migraine Diagnosis Duration (in years), mean (SD)**	19.2 (16.2)
Medication, N (%)*	
Non-prescription	42 (24.7%)
Prescription to treat migraine	90 (52.9%)
Prescription drug to avoid migraine	33 (19.4%)
Other (e.g., homeopathic medication, vitamins)	5 (2.9%)
**Monthly Migraines mean (SD), median, [min, max]**	5.22 (4.1), 4, [1,20]

* Options not mutually exclusive; therefore, patients can fall into several categories.

### Headache-related disability

Almost half of the participants (51 out of 106) could be categorised as suffering the highest level (Grade IV) of migraine-related disability, based on their MIDAS scores in the past three months. The number of participants that could be categorised as Grades I, II, and III was 9, 14, and 32, respectively. The mean (SD) number of lost days (due to either missed work or impaired productivity) in the last three months due to headache for Grades I, II, III, and IV of disability was 2.78 (2.44), 7.79 (1.53), 15.31 (2.72), and 48.00 (29.55), respectively.

#### Comparison of utility values between severity levels, current health (without migraine), and "no problems on any EQ-5D dimension"

Utility values were estimated based on data from all 106 participants. One participant did not complete the EQ-5D for their current health (without migraine), and therefore EQ-5D data for current health (without migraine) was available for 105 participants. In addition, 11 participants did not experience any period of mild migraine pain, 16 participants did not experience any period of moderate migraine pain, and 37 participants did not experience any period of severe migraine pain. Therefore, data for mild, moderate, and severe levels of migraine pain severity were available for 95, 90, and 69 participants, respectively.

Table 2 shows the analysis of mean EQ-5D utility scores for each health state, and demonstrates that each health state utility value was found to be significantly (p < 0.0001) different from 1.0 (no problems on any EQ-5D dimension). The mean utility score for current health (without migraine) was 0.87 (0.15 SD), and was the highest utility score from among the four health states (current health without migraine, mild migraine pain, moderate migraine pain, and severe migraine pain).

**Table 2:** Analysis of Mean EQ-5D Utilities and Disutilities and Comparison to a Value of 1.0 ("no problems on any EQ-5D dimension")

**Level**	**N**	**Mean Disutility = (1 – Utility)**	**Health Utility**				
			**Mean Utility**	**SD**	**Lower 95% CI**	**Upper 95% CI**	P Value*
Current health without migraine	105	0.13	0.87	0.15	0.84	0.90	<0.0001
Mild	95	0.34	0.66	0.23	0.62	0.71	<0.0001
Moderate	90	0.47	0.53	0.27	0.47	0.59	<0.0001
Severe	69	1.20	-0.20	0.29	-0.27	-0.13	<0.0001

* P-values resulting from the one-sided *t*-test using the null hypothesis that mean equals 1 (i.e., "no problems on any EQ-5D dimension").

Utility values were lower for more severe levels of migraine pain, with the lowest mean utility score of -0.20 (0.29 SD), obtained for a severe level of migraine pain. Mild and moderate levels of migraine pain severity had mean utility values of 0.66 (0.23 SD) and 0.53 (0.27SD), respectively.

#### Comparison of utility values between health states

**Figure 1:**
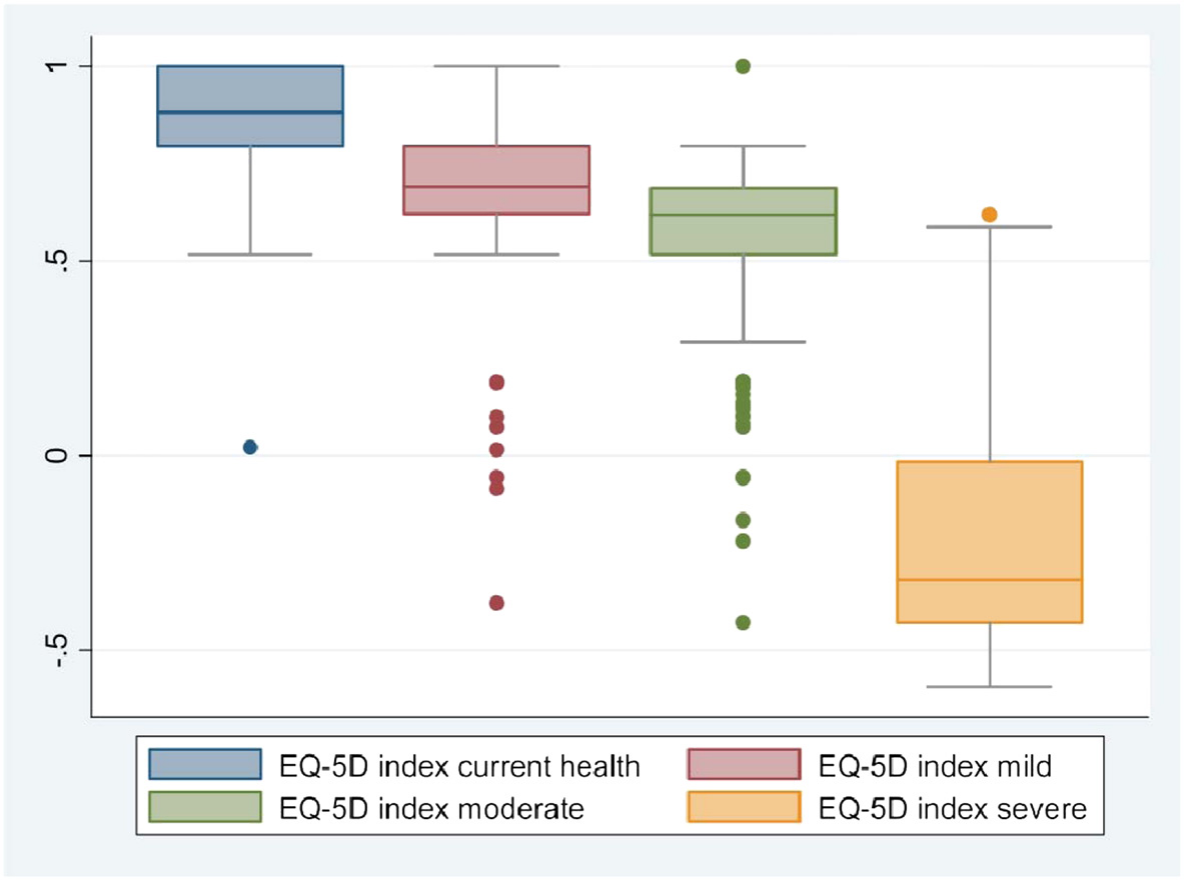
Utility of Each Health State Showing the Median and Inter-quartile Range.

Figure 1 illustrates the median scores for the EQ-5D utility values obtained for each health state and the 25th and 75th percentile for each health state and shows that as severity level worsened, utility scores decreased.

We compared mean EQ-5D values for each health state with one another to examine whether utilities were different among the migraine pain severity levels (Table 3). Note that sample sizes for each comparison vary, based on the number of respondents who reported having experienced both health states. Parametric test (paired *t*-test) was used to compare mean utility values between each level of severity to evaluate whether there were significant differences in mean utility scores by migraine severity. Further testing of the distribution patterns for the EQ-5D data at each health state indicated a slight departure from normal distribution, and thus parametric tests may be biased. Therefore, a non-parametric (Wilcoxon signed rank) test was performed. However, results of Wilcoxon signed rank test were consistent with the results of the parametric tests.

**Table 3:** Difference in Utility Among Health States

	**Mild**	**Moderate**	**Severe**	**Current Health**
	**0.66**	**0.53**	**-0.20**	**0.87**
Mild	--			
Moderate	0.13^1^	--		
Severe	0.86^1^	0.73^1^	--	
Current Health	0.21^1^	0.34^1^	1.07^1^	--

^**1**^ P < 0.0001 using parametric (paired *t*-test) and non-parametric (Wilcoxon signed rank) tests.

Results indicate mean utilities for mild, moderate, and severe migraine pain were significantly different from one another (p < 0.0001 for each comparison). The mean utility for each health state (including mild migraine) was significantly worse than the utility of the current health (without migraine) state (p < 0.0001). The smallest difference in mean utility scores was between mild and moderate migraine pain severity levels (0.13), and the largest difference in mean utility scores was between current health (without migraine) and severe migraine pain severity (1.07).

### Headache-related disability and associated utility

Significant negative correlations between current health (without migraine) utility and MIDAS scores were found (r = -0.29, p < 0.003); as such, higher MIDAS scores (more disability due to migraine) were associated with lower current health (without migraine) utility values. Correlations were not very high, but were statistically significant at the 1% level.

## Discussion

The results of this observational study with participants who had recently experienced a migraine in the UK suggests that utility scores for each migraine pain health state were significantly different from 1.0 (no problems on any EQ-5D dimension) (p < 0.0001) and one another (p < 0.0001); with severe migraine pain estimated to have the lowest mean utility, -0.20 (95% confidence interval [CI]: -0.27 – -0.13), this is valued as a state worse than death.

This study reports EQ-5D-derived utility values for mild, moderate, and severe migraine pain levels of a recent migraine attack. EQ-5D data for a recent migraine attack was gathered based on data collected from migraine sufferers in the UK within seven days of the most recent attack.

### Impact on health status

The mean utility of current health (without migraine) in this UK study (0.87) was similar to the EQ-5D utilities elicited in a study of US migraineurs (sampled either outside of or during an attack) estimated at 0.82 by Luo et al. (2009) [13]. Statistically significant differences (p < 0.0001) were also found between 1.0 (no problems on any EQ-5D dimension) and each level of migraine pain severity, and also between current health (without migraine) and every level of migraine pain severity (p < 0.0001)—indicating that migraine significantly and negatively affects participants’ utility.

We also found significant negative correlations between EQ-5D utility for current health (without migraine) and MIDAS scores. This suggests that patients’ experience of migraine pain-related disability may significantly and negatively affect their utility, with greater migraine-related disability associated with lower health utilities.

### Migraine pain utilities and utilities for other disease areas

We found that differences in utility for moderate and severe migraine pain and current health without migraine (0.34 and 1.05, respectively) were greater than those found for a decrease in health utility following *hip fracture* when compared to pre-fracture, as measured using the EQ-5D (0.12-0.20) [18,19], and utility decrements of 0.13 and 0.14 for *rheumatoid arthriti*s and *hand osteoarthritis*, respectively, as measured using the SF-6D [20]. Xu et al. (2010) [12] reported similar findings.

### Health status by severity

This study further demonstrated that the levels of severity of migraine examined here are tangible: patients were able to value different levels of migraine pain severity of a recent migraine attack.

While this study aimed to examine the utility values only for migraine pain, participants may have considered other aspects of their migraine headaches, such as nausea and sensitivity to light and sound. However, previous studies have generally found a positive association between the severity of migraine pain and the accompanying symptoms of migraine [21,22], and the migraine-specific triptan class of medications has been shown to relieve these symptoms along with reducing the severity of migraine pain [23]. Thus, the extent and directionality of any bias in the practical application of these utility values to therapeutic interventions for migraine is unclear, to the degree that treatment benefits also encompass these symptoms.

The results of our study suggest that having a migraine results in a significantly reduced utility (health state preference), with more severe migraine pain resulting in lower utility. Other studies have yielded similar results. Xu et al. (2010) [12] also found that EQ-5D-derived utility worsened with increasing migraine pain severity. However, unlike Xu et al. (2010) [12] who found the disutility of mild and moderate migraine to be relatively similar in magnitude (0.14 vs. 0.18), our study indicates a statistically significant difference of nominally greater magnitude between the disutility for mild (0.20) and moderate (0.34) migraine. Our analysis also found that, while eliminating a migraine yields the greatest improvement in health utility, the difference between moderate and mild severity in our analysis was significant, suggesting that reducing migraine severity to a mild would still be of value to migraine sufferers. These different results may be due to differences in study design.

Disutility values relative to current health without migraine estimated from this study for mild, moderate, and severe levels of migraine pain were higher (0.21, 0.34, and 1.07, respectively) than those estimated by Xu et al. (2010) [12]: 0.14, 0.18, and 0.49, respectively. This may also be a result of the methods used to obtain utilities in the respective studies. Patients in Xu et al.’s (2010) [12] study were evaluated while experiencing moderate/severe migraine pain at the baseline of the clinical trial, within 24 hours of first treating a migraine attack, and 24 hours later when the participants were pain free. By contrast, patients in our study were evaluated within seven days of a migraine attack, while they experienced no residual symptoms. Also, Xu et al. (2010) [12] applied the scoring algorithm for the US population [24], whereas the present study used the UK population scoring algorithm. The same health state tends to be valued more severely in the UK. For instance, the worst health state was scored as -0.11 using the US algorithm, which was higher than the UK valuation for severe migraine pain reported in this study (-0.20). These observed differences may also be a result of the context within which utilities were elicited (clinical trial vs. naturalistic, observational study) and differences in sample characteristics; for example, a higher percentage of females and participants of white or Asian origin, and a lower percentage of participants of black origin and no participants of Hispanic origin in our sample, as compared with Xu et al. 2010 [12].

The reported utility value for severe migraine that is "worse than dead" (which is in the range of the EQ-5D utility scale) is based on time-tradeoff utilities for members of the UK general public who evaluated profiles for the underlying EQ-5D health states corresponding to those selected by the migraine patients in our study when they considered the severe level of pain of their recent migraine. In ascribing a utility value of worse than dead, the members of the UK general public provided valuations for states regarded as worse than dead by making a choice between dying immediately or spending a length of time (10-x) in the target state, followed by x years in the 11111 state (no health problems on any domain) and finding the point of indifference. The utility score was given by the formula -x/(10-x) [6]. Thus, these states were regarded by members of the UK general public as being less desirable than a loss of life for that time span.

Another analysis eliciting migraine-related utilities from a US adult patient sample with 4-week recall (potentially covering time with and without migraine) using the HUI3 found migraine severity (self-assessed on a scale of 1 to 10) was not a significant predictor of utility [11]. However, authors note that this lack of a relationship between migraine-related pain and utility may have been due to the low variation in pain seen in the dataset.

### Limitations

Some limitations should be noted with respect to our analysis. Deciding on the optimal recall period in which a participant can and should reflect on their experiences is complex; and there is a large amount of literature which demonstrates that memory bias (such as confounding or over/under estimating experience) is inherent in the task of recalling complex information retrospectively [25,26]. This may be particularly true for the recall of pain [27]. Some design features of the current study (e.g., subjects were not primed to report on dates and hours of their recent migraine) may have resulted in such memory bias, and therefore error.

We reported data for a convenience sample of migraineurs who were willing to participate in the study. Data on geographical distribution was not collected. It was not a random sample and may not be representative of the UK migraine population as a whole. The mean age and gender distribution of our sample appears similar to that reported from a national sample of English migraineurs aged 16-65; however, the monthly attack frequency within our study population was higher (five vs. two attacks per month), along with the proportion of non-Caucasians [28]. We were also not able to obtain EQ-5D-derived utilities during a migraine attack. We collected this data retrospectively within a week of participants’ most recent migraine, and it is possible that the number of migraines experienced over the retrospective period would have influenced the recall of migraine pain severity. For example, recall may be different in participants who suffer many migraine attacks in a given four-week period, compared with those who experience relatively few. Bias may exist in participants’ recall of migraine-related disability and the effect of their migraine pain on their HRQL (as more recent experiences are likely to be recalled differently than those happening further in the past). It may also be important to assess intra-patient mean differences rather than group mean differences, where the latter may not enable inference of the population as whole.

Utility weights for EQ-5D index health state profiles have been previously estimated via the TTO method based on a 10-year timeframe [6], while individual migraines are of shorter duration (4-72 hours). However, as noted by Xu and colleagues [12], when modelled over a patient’s lifetime, the cumulative duration of episodes of migraine is likely to be within the range of length of many other conditions which are readily valued using the EQ-5D (e.g., treatment for various cancers, osteoporotic fracture).

Finally, it is also important to note that this study does not directly examine the utility associated with improved migraine management through treatment, and if used in economic analyses of migraine therapies, the assumption would be that any utility benefits observed are generalisable to those obtained with a treatment that reduces the number or severity of migraine hours experienced.

## Conclusion

Migraineurs attribute different values to various levels of migraine pain severity. In this UK sample, using the EQ-5D, we found that greater migraine pain severity was associated with lower utility. Having a migraine resulted in significantly reduced utility regardless of pain severity, with utility decreasing as pain severity increased. Significant differences were found in utility among all health states evaluated, and in the utility of each health state evaluated compared with 1.0 ("no problems on any EQ-5D dimension"). While results from this study indicate elimination of migraine could yield the greatest improvement in utility, results also suggest that reducing migraine pain severity (from severe to moderate or from moderate to mild) could also result in a significant improvement in utility. Results from this analysis can be useful for evaluating the relative value of therapies used to treat migraine, and may be of particular use in cost-effectiveness and cost-utility models evaluating the migraine treatment landscape in the UK.

Differences in methods used to estimate utilities (e.g., patient vs. population preferences, type of survey, direct vs. indirect estimation) and contextual factors (e.g., geographical setting, clinical trial- vs. community-sample) can influence utility values, and should be considered when using utilities from various sources for a single cost-utility model.

## Abbreviations

CI, Confidence interval; EQ-5D, EuroQol; EQ-5D, Survey; HRQL, Health-related quality of life; HUI, Health Utilities Index; HUI2, HUI Mark 2; HUI3, HUI Mark 3; MIDAS, Migraine Disability Assessment Questionnaire; NICE, National Institute for Health and Clinical Excellence; QALY, Quality-adjusted life year; SD, Standard deviation; SG, Standard gamble; TTO, Time trade-off; UK, United Kingdom; US, United States; VAS, Visual Analogue Scale.

## Competing interests

This study was funded by Merck Sharp & Dohme, Corp. Ralph Insinga (RPI) and Ruifeng Xu (RX) are employees of Merck Sharp & Dohme, Corp.

Daisy S. Ng-Mak (DSN) was an employee of Merck Sharp & Dohme, Corp. at the time of first submission.

Megan Stafford (MRS), Asha Hareendran (AH), and Donald Stull (DS) were employees of United BioSource Corporation and served as paid consultants to Merck Sharp & Dohme, Corp. during the conduct of this study and the development of this manuscript.

## Authors’ contributions

DSN, AH, RPI, RX, and MRS have made substantial contributions to conception and design, acquisition of data, or analysis and interpretation of data; MRS, AH, DSN, RPI, and RX have been involved in drafting the manuscript or revising it critically for important intellectual content; and all authors have given final approval of the version to be published. All authors read and approved the final manuscript.

## Authors’ information

Merck continues to be committed to migraine therapy.
